# A needle-to-post air discharge ion source in tandem with FAIMS system

**DOI:** 10.1371/journal.pone.0221080

**Published:** 2019-08-16

**Authors:** Hua Li, Hongmei Yun, Yongrong Jiang, Ruosheng Zeng, Zhencheng Chen

**Affiliations:** School of Life and Environmental Sciences, GuiLin University of Electronic Technology, Guilin, Guangxi, China; Fisheries and Oceans Canada, CANADA

## Abstract

A needle-to-post ionization source was designed for high-field asymmetric waveform ion mobility spectrometry (FAIMS). The needle-to-post ion source includes asymmetric electrode comprised of a copper post with a diameter of 2 mm and a stainless-steel needle with 200-μm tip radius and length of 28 mm. With the discharge voltage of -5.6 kV and N_2_ gas flow, glow discharge was realized at atmospheric pressure. The mass spectra of ionized ions about acetone, ethanol and ethyl acetate were gotten by Thermo Scientific LTQ XL ion trap mass spectrometer (MS). The MS experimental results show that the main ions are protonated and dimer ions. The needle-to-post ion source was mounted on the FAIMS system and FAIMS spectra are gotten successfully. Separation of p-xylene, o-xylene and m-xylene was realized. It shows that the needle-to-post electrode could be used as the ion source in a FAIMS system.

## 1.Introduction

High-field asymmetric waveform ion mobility spectrometry (FAIMS) enables the separation and determination of ions by differences in ion mobility at high and low field strength [1–3]. It has been widely used because of its high sensitivity, low power consumption, and rapid detection.

The FAIMS system consists of three main parts: an ionization region, a drift tube, and a detection region. An important component is the ion source, which ionizes the sample for subsequent detection and analysis [4]. Until now, six kinds of ion sources have been used in FAIMS. UV-lamp ion source was most widely used in FAIMS system for its simple structure and the simple ion species as a soft ionization method [5]. Meanwhile, the ionization energy of photoionization sources (10.6eV) is limited; therefore, they cannot ionize substances that exceed this ionization energy. G. A. Eiceman et al adopted radioactive ionization source (^63^Ni) for planar field asymmetric ion mobility spectrometer [6]. Radioactive ionization sources require specific licenses and have associated risks. Roger Guevremont et al used ESI (electrospray ionization) for cylinder FAIMS system [7]. Electrospray ion sources have been used widely in mass spectrometers and are generally suitable for biomacromolecule and substances with strong ionization polarity. ESI is a soft ionization and can get stable molecular ion peak. For FAIMS system, there are also shortcomings with ESI ion source. ESI ion source needs solvent, which bring the ion-molecule reaction and hard to be separated. Then ESI ion source is seldom adopted in planar FAIMS system. In recent years, some kinds of new ion sources are adopted used in FAIMS. Andriy Kuklya designed a low-temperature plasma ionization and X-ray ionization for FAIMS in 2015 and 2017, respectively [8–9]. The commercialization of X-ray limits its widely use in FAIMS experiment. A needle-mesh corona discharge was used by Zhao et al. to detect dimethyl methylphosphonate in 2013 [10]. Discharge characteristics and ion species have not been reported. Furthermore, direct comparison of the gas discharge with other ion sources for FAIMS is also needed.

Despite the use of gas discharge ion sources for FAIMS, no discharge characteristics and the ion species were reported. Furthermore, a direct comparison of the gas discharge with other ion sources for FAIMS needs to be performed.

In our previous study, a needle-to-post chip device was reported [11–12]. The chip device has a needle and a pair of post arrays. When a negative direct current voltage was applied to the needle and post electrodes, corona and glow discharge were generated, respectively. Correspondingly, it can ionize the chemical samples. But the ion species are not gotten. And the question whether it can work stably as an ion source for FAIMS is not answered in the former paper.

Here, a new needle-to-post ion source was designed for FAIMS. Acetone, ethanol, and ethyl acetate standard samples were analyzed. An analysis of the FAIMS spectra acquired with a needle-to-post ion source indicated that the asymmetric electrode structure for the ionization source was feasible. Mass spectrometry with a needle-to-post ion source was designed to operate at atmospheric pressure and room temperature, and its performance was compared with that obtained with a UV lamp ion source.

## 2. Experimental

**Fig 1:**
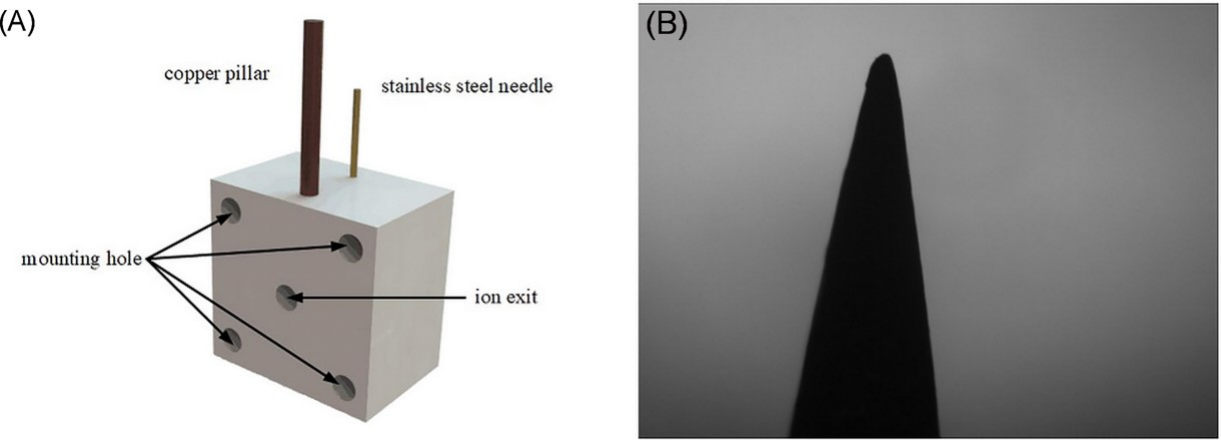
Needle-to-post ion source. (A) Peripheral device for the needle-to-post electrode; (B) Photograph of the tip curvature.

Fig 1A shows the structure of the needle-to-post ion source, which used an asymmetric electrode comprised of a copper pillar (or post) with a diameter of 2 mm and a stainless-steel needle with a length of 28 mm. The post was the discharge anode, while the needle was the discharge cathode at a 8-mm gap distance. The 200-μm tip radius of the needle was measured with an optical microscope (OPTPro 2008) and image processing, as shown in Fig 1B.

After ionization, the ions entered the FAIMS through a 3-mm circular exit port. The ion source was mounted in the FAIMS via machined holes. The overall size of the needle-to-post ion source was 28-mm×28-mm×15-mm.

**Fig 2:**
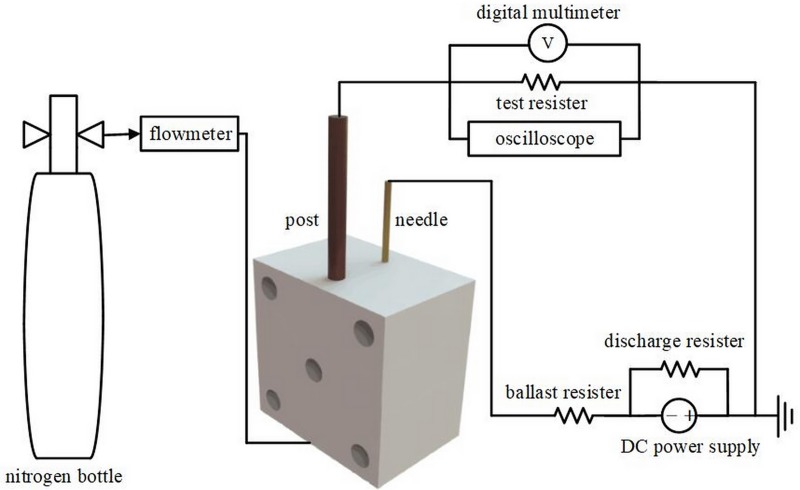
Schematic of needle-to-post discharge circuit.

The discharge circuit (see connection block diagram in Fig 2) of the needle-to-post ion source consisted of a DC power supply, a discharge resistance, and a ballast resistor, and was powered with a 0–-20-kV, 100-W DC power supply. The discharge resistor released electric charge when the power supply voltage was decreased. The 12-MΩ ballast resistance was connected to the needle to restrict the discharge current. A 1-kΩ test resistor converted the discharge current into voltage, which was measured with an oscilloscope and a digital multimeter. The oscilloscope (Tektronix) was used to observe and record the actual AC discharge voltage, whereas the multimeter was used to measure the specific voltage.

A carrier gas is needed when using the needle-to-post ionization source. The effect of carrier gas flow on the discharge characteristics of the source was examined at different flow rates. The carrier gas was pure nitrogen (99.999%) purchased from Beijing Hua Yuan Gas Chemical Industry Corporation.

**Fig 3:**
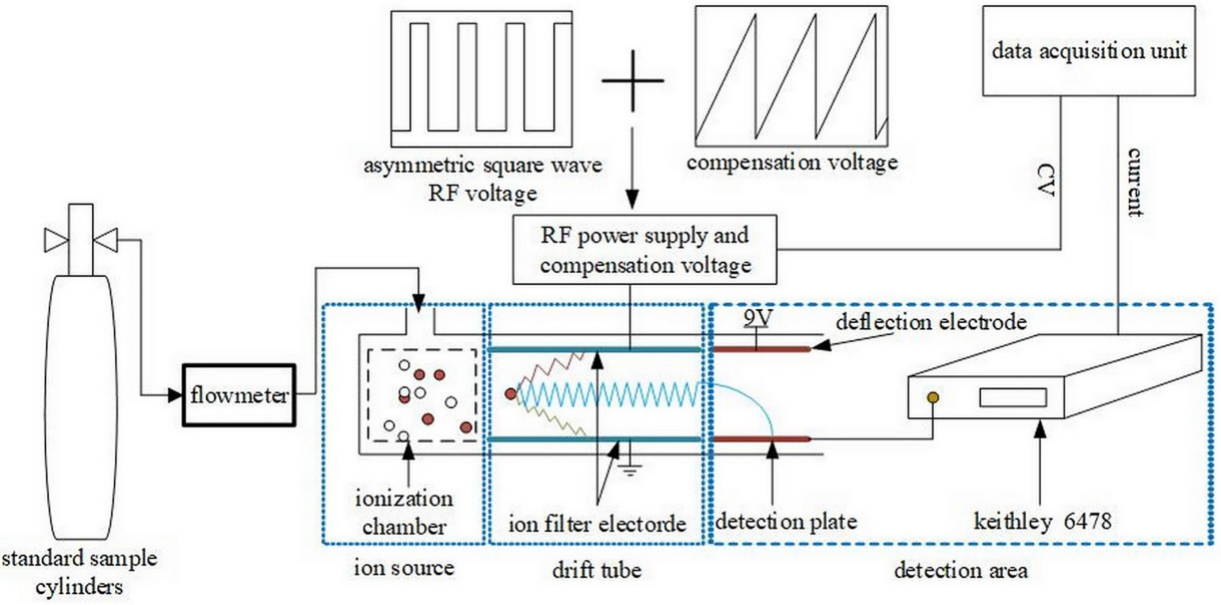
Structure of FAIMS system.

The home-made FAIMS, described previously [13], is schematically depicted in Fig 3.

The spacing of drift tube was 0.2 mm. An asymmetric square wave RF voltage was used to separate the ions, which were then collected by the detection electrode. The ion current and the corresponding compensation voltage were recorded simultaneously, which was used by LabView software to realize the FAIMS spectrum of the sample in real time. The FAIMS performance characteristics of the needle-to-post and UV-lamp ion sources (Heraeus, PKS106) were compared.

Samples (Table 1) were supplied by a high-pressure standard gas bottle (Beijing Huayuan Gas Chemical Co., Ltd.). The flowmeter model was a D08-1F provided by Beijing QixingHuachuang Electronics Co., Ltd. The 1-MHz asymmetric square waveform (30% duty cycle) and the compensation voltage (-10 V to +10 V) were applied to the drift tube. The amplitude of the asymmetric square waveform varied over 400–700 V.

**Table 1:** Gas samples used in FAIMS experiment.

Samples	concentration
Acetone	99.7 μg/ml
Ethanol	300 μg/ml
Ethyl acetate	99.3 μg/ml

**Fig 4:**
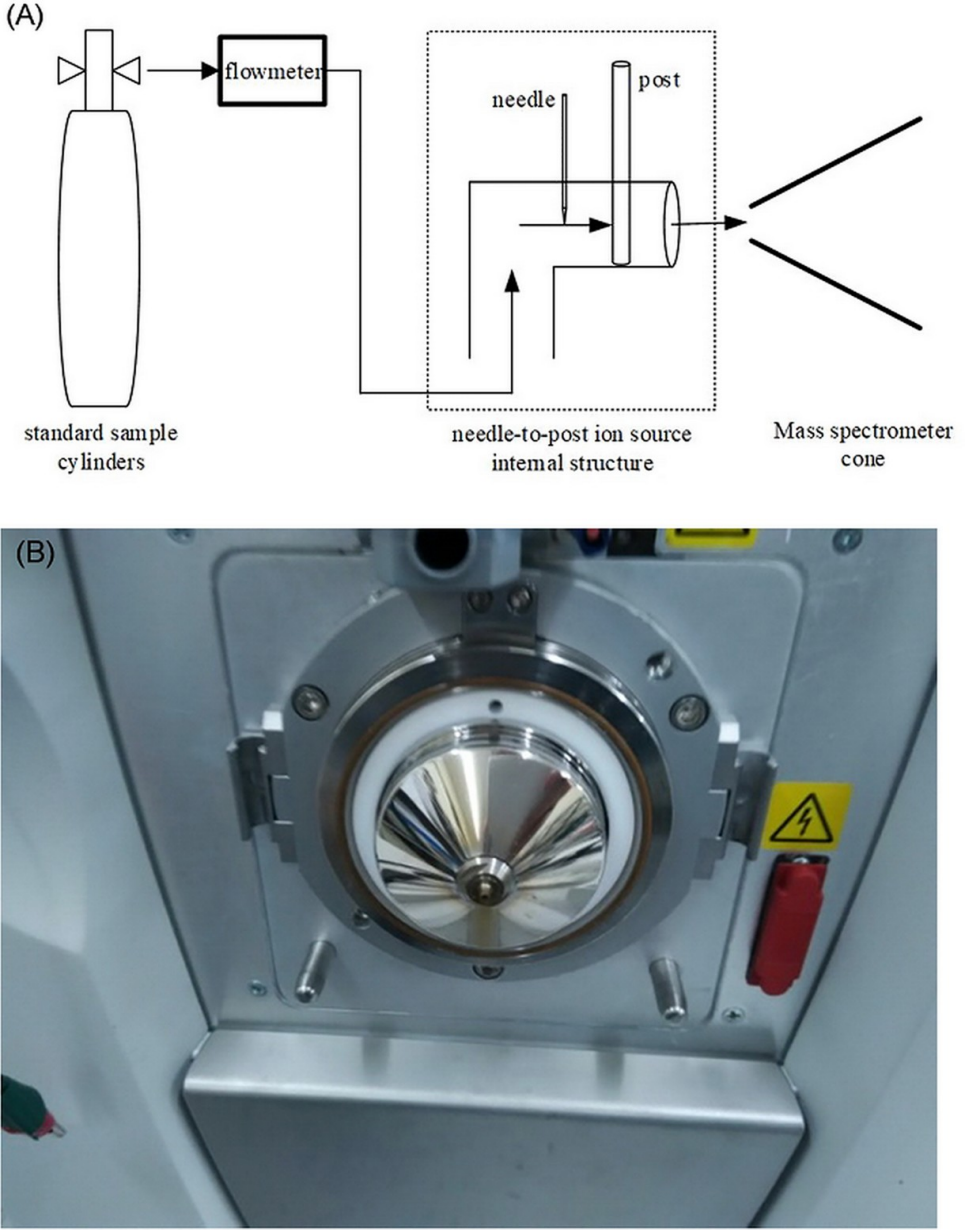
Block diagram of needle-to-post ion source for mass spectrometry. (A) Schematic of experiment system; (B) Thermo Scientific LTQ XL ion trap mass spectrometer.

A schematic of the device structure for the mass spectrometric analysis is shown in Fig 4.

The chemical accuracy of the needle-to-post ion source was verified by comparison of mass spectra using the UV lamp ion source. The experiments used a Thermo Scientific LTQ XL ion trap mass spectrometer at a temperature of 30°C, and a tapered voltage of 0 V, which is equipped with ESI. In the experiment, the ESI ion source was removed. Then the needle-to-post ion source and UV-lamp ion source is installed. The gas samples used for the mass spectrometric analysis experiments were same as those used for the FAIMS experiments.

### 3. Experimental results and discussion

#### 3.1 Discharge characteristics of the needle-to-post ion source

**Fig 5:**
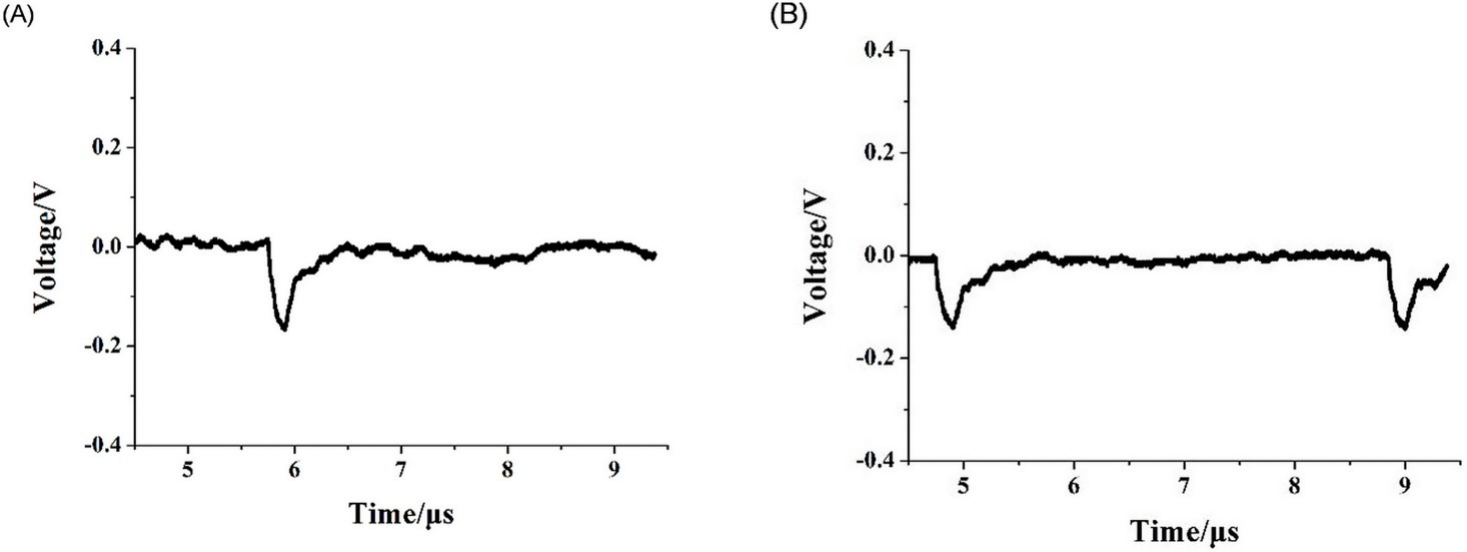
Discharge waveforms for needle-to-post electrode for given discharge voltages. (A) the discharge voltage of -4.9kV; (B) the discharge voltage of-5.6 kV.

The needle-to-post ion source discharge was studied at room temperature and atmospheric-pressure without external gas flow. Discharge waveforms recorded by the TDS2001C oscilloscope under different voltage discharge conditions are shown in Fig 5.

Fig 5 shows a typical Trichel pulse, indicating that the needle-to-post structure ion source was in a stable corona discharge stage. With increasing discharge voltage (-5.6 kV), the Trichel pulse frequency also increased, but the pulse amplitude decreased. Meanwhile, the discharge current measured with the digital multimeter increased with the discharge voltage. The similar experimental results were also reported in our former published papers and other related literatures [11–13]. Because the needle-to-post discharge space is relatively small, the source rapidly enters the spark discharge stage.

**Fig 6:**
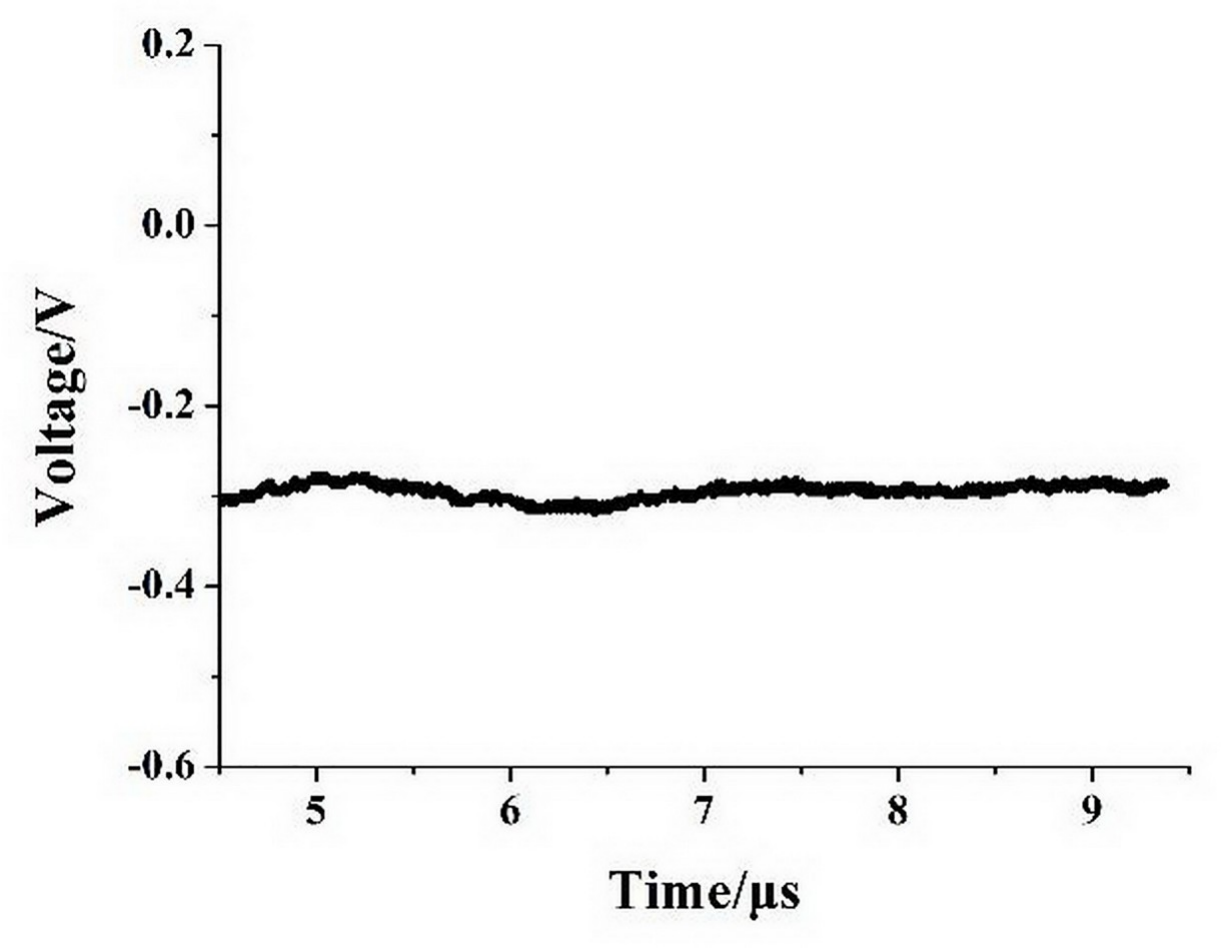
Discharge waveform of needle-to-post ion source, glow discharge at -5kV, and a carrier gas flow rate of 1.5 l/min.

When the carrier gas was introduced at a certain flow rate, the waveforms generated by the needle-to-post discharge were recorded by the oscilloscope, as shown in Fig 6.

The Trichel pulse disappeared completely, leaving only a DC voltage. According to the waveform, the needle-to-post ion source directly entered the glow discharge stage after the carrier gas was introduced. Also, the direct voltage increased with increasing discharge voltage, and the discharge current measured by the digital multimeter increased with the voltage.

**Fig 7:**
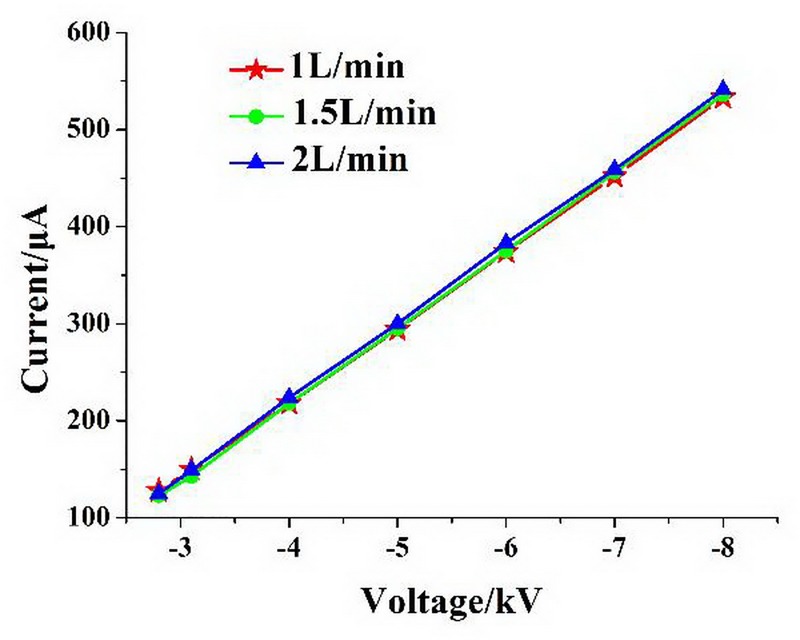
Glow discharge voltage-current characteristics under different carrier gas flow rates.

Gas flow rates of 1 L/min, 1.5 L/min, and 2 L/min were used. The V-I characteristics of the needle-to-post ion source as a function of flow rate are plotted in Fig 7.

The discharge current *I* increases linearly with increasing discharge voltage *V* under different carrier gas flow rates. Thus, the carrier gas flow rate has little effect on the discharge current.

### 3.2 FAIMS experiment

The needle-to-post ion source was mounted on the FAIMS system as shown in Fig 3. Experiments under different conditions were recorded when the stable glow discharge of the gas sample was formed between the needle and the post.

#### (1) Effects of the carrier gas flow rate

**Fig 8:**
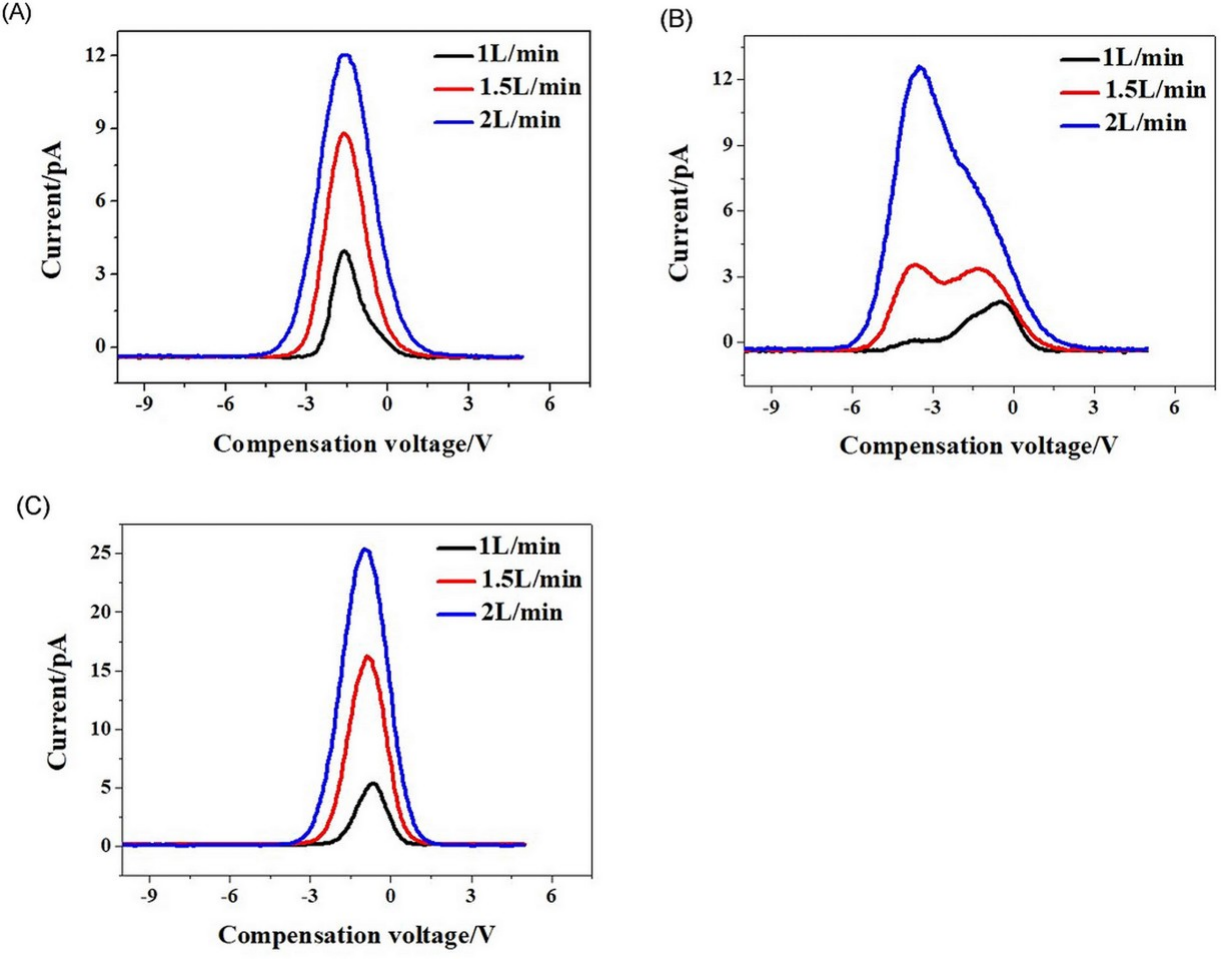
FAIMS spectra of three samples at different carrier gas flow rates, at a 400-V RF voltage and a -8-kV discharge voltage. (A) Acetone; (B) Ethanol; (C) Ethyl acetate.

The carrier gas flow rate was controlled by the flowmeter. When the carrier gas flow rate was changed, the FAIMS spectrum also changed. For a -8-kV discharge voltage, FAIMS spectra of three samples at different carrier gas flow rates are shown in Fig 8.

By comparing Fig 8(A)–8(C), it can be seen that the corresponding compensation voltages of the FAIMS spectra were not the same for the different samples. For each same sample, the ion signal intensities increased and the spectra broadened with increasing gas flow. The ion signal strength *I*_*st*_ for plate-type FAIMS is given by [14–15]:

$$I_{st}=n_{in}\times Qexp\left[-\frac{\pi^{2}k_{B}KTt_{res}}{e(d-\frac{2KV_{pk}D}{fd})}\right]$$ (1)

where *n*_*in*_ is the ion concentration at the entrance of the drift tube, *Q* is the carrier gas flow rate, *k*_*B*_ is Boltzmann’s constant, *T* is the temperature (K), *t*_*res*_ is the time of migration of the ions through the drift tube, *e* is the number of ion charges, *d* is the migration area of the plate spacing, *f* is the asymmetric square-wave frequency with duty cycle *D*, *V*_*pk*_ is the peak-to-peak amplitude of the asymmetric square wave signal, and *K* is the ion mobility under high field conditions. When *Q* is increased, with other conditions unchanged, Eq 1 indicates that *I*_*st*_ increases. The results show that the FAIMS experiments were in agreement with the theory, in contrast to the FAIMS experiments with the former ion source.

The FAIMS system resolution *R* and the plate-type FAIMS spectral half width are given by [16]:

$$R=\frac{V_{cv}}{W_{1/2}}$$ (2)

$$w_{1/2}=\frac{Qd}{hLK_{0}}H$$ (3)

where *V*_*cv*_ is the compensation voltage, *h* is the lateral area of the drift tube, *L* is the length of the drift tube, *K*_0_ is the ion mobility under low-field conditions, and *H* is the relative peak height of the FAIMS spectrum. The half-peak width of the FAIMS spectra is the distance between two points where the current value of the ion signal is half of the peak.

When the carrier gas flow rate increases and other conditions remain unchanged, then *w*_1/2_ increases, according to Eq (3). Then, from Eq (2), we know that *R* is reduced. Hence, an increased carrier gas flow rate will broaden the FAIMS spectrum, reducing the resolution of the detection system.

#### (2) Effects of discharge voltage

A high-voltage DC power supply (0–-20 kV) was connected to the needle-to-post ion source. In the FAIMS experiment, the discharge voltage value between the needle and the post was increased, while the carrier gas flow rates were constant at 1.5 L/min.

**Fig 9:**
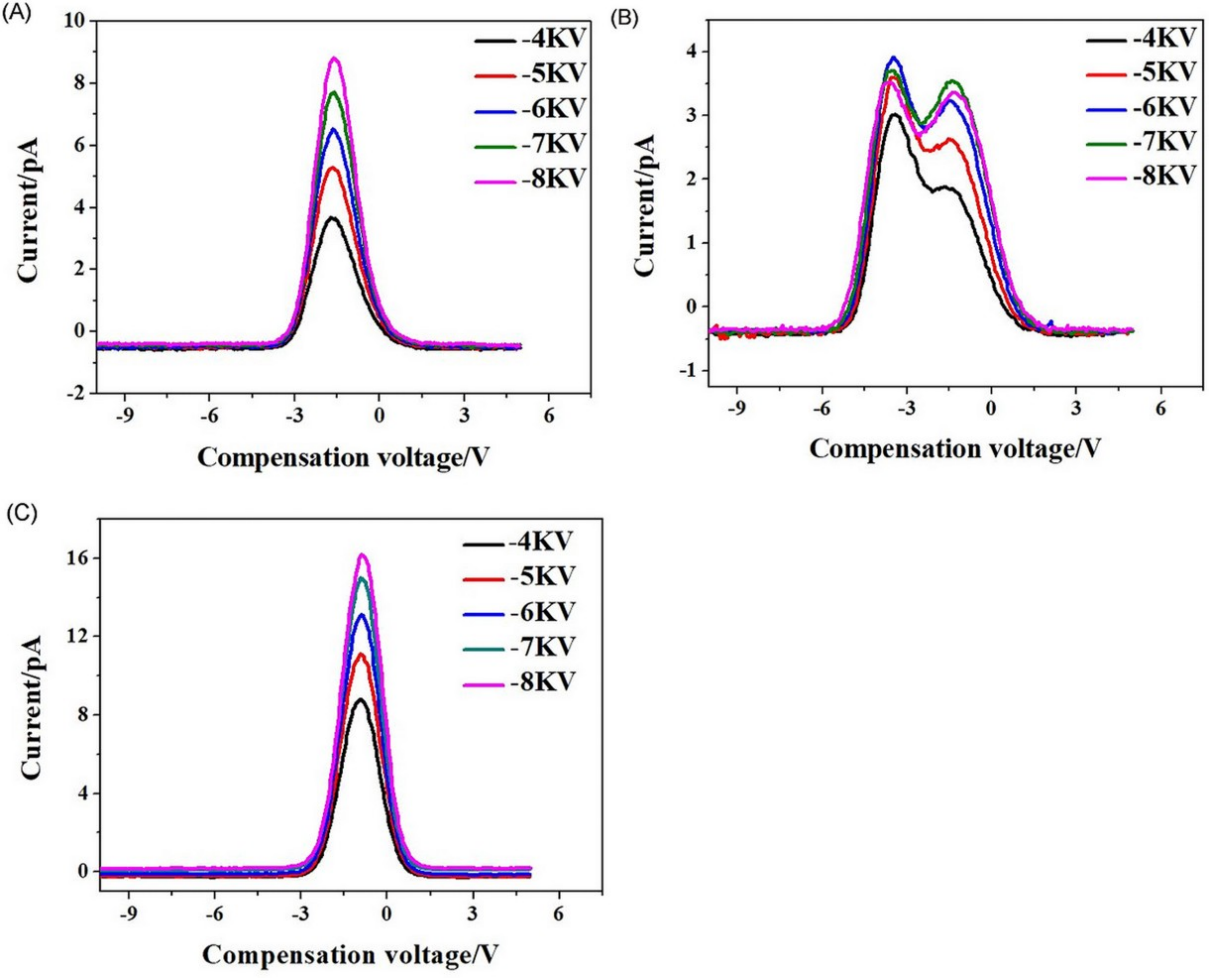
FAIMS spectra of three samples at different discharge voltages. (A) Acetone, *Q* = 1.5 L/min; (B) Ethanol, *Q* = 1.5 L/min; (C) Ethyl acetate, *Q* = 1.5 L/min.

FAIMS spectra of acetone, ethanol, and ethyl acetate samples as a function of discharge voltage are shown in Fig 9.

The ionic signal strength in the spectra increased with the discharge voltage because the discharges between the needle and the post were more intense at increased voltages, generating more ions. In the plate-type drift tube, the increased number of generated ions increased the ion concentration *n*_*in*_ at the entrance of the drift tube. According to the Eq (1), when the other parameters remain constant, the ionic signal intensity of the FAIMS spectrum increases with *n*_*in*_. For acetone and ethyl acetate, the spectra followed the theory. But the other interesting experimental result shows that the change law is not obvious during the discharge voltage -5kV~-8kV for the ethanol spectra. The possible reason is that the ionization efficiency for ethanol is almost saturated during -5kV~-8kV.

#### (3) Effects of *V*_*pk*_

**Fig 10:**
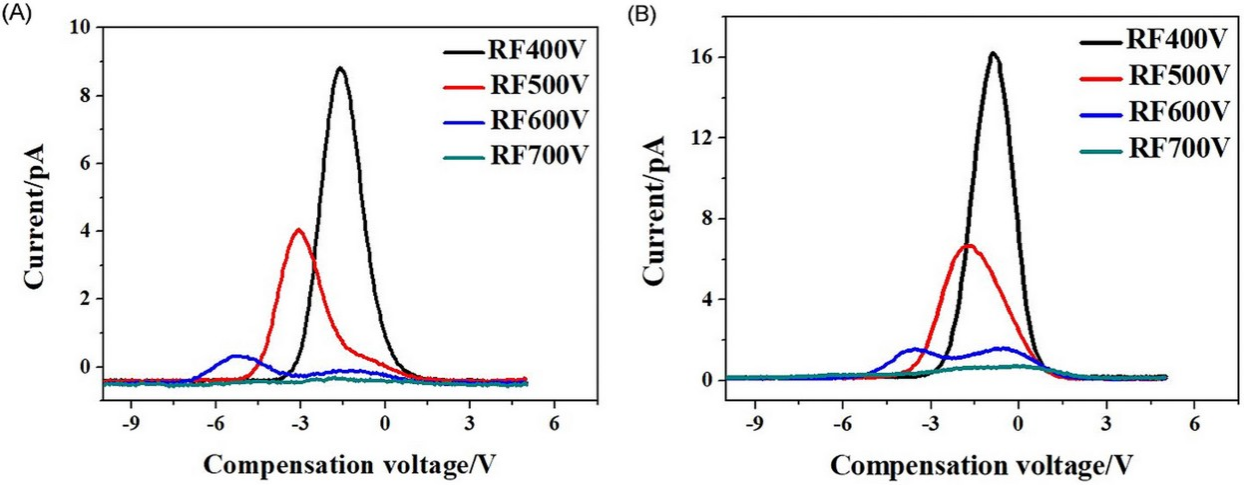
FAIMS spectra of three samples at different RF and -8-kV discharge voltages. (A) Acetone, *Q* = 1.5 L/min; (B) Ethyl acetate, *Q* = 1.5 L/min.

Changing the peak-to-peak voltage *V*_*pk*_ of the asymmetric square wave signal had an effect on FAIMS spectra, as shown in Fig 10 for the three samples. The discharge voltage was -8 kV for the needle-to-post ion source and the carrier gas flow rate was 1.5 L/min.

According to Eq (1), when other parameters remain constant, the ionic signal intensity of FAIMS spectra will decrease as *V*_*pk*_ increases. Under constant carrier gas flow rate and discharge voltage, FAIMS spectra of acetone and ethyl acetate indicated that increasing *V*_*pk*_ reduced the ionic signal strength of the spectra, which was consistent with the theory. In addition, the value of the compensation voltage increased with *V*_*pk*_, and there was more than one peak when *V*_*pk*_ increased. For example, two peaks appeared for *V*_*pk*_ = 600 V, but there was only one peak for *V*_*pk*_ = 400 *V*. Thus, the resolution increased with *V*_*pk*_.

#### (4) Separation of isomers

P-xylene, o-xylene and m-xylene are the typical isomers. Xylene isomers have similar physical and chemical properties because they have the same molecular weight (*M* = 106 amu) but the different structure formulas. They are difficult to be separated by IMS.

**Fig 11:**
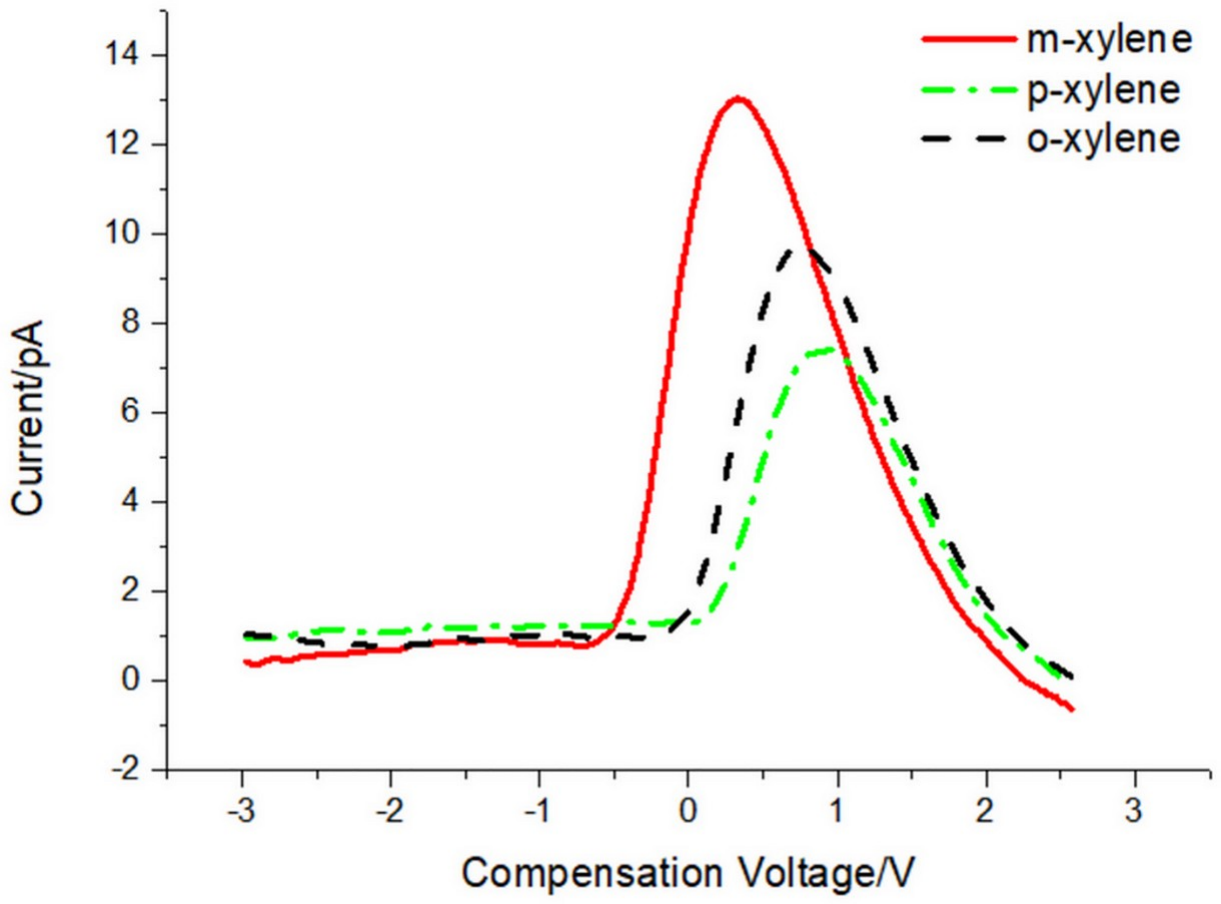
FAIMS spectrum of m-, o- and p- xylene.

Under 400 V RF voltage and 2 L/min gas flow rate, the separation results of xylene isomers (Shanghai Macklin Biochemical Co., Ltd, AR 98%) are shown in Fig 11.

To get the right result, every experiment is repeated three times to ensure the repeatability. The compensation voltage of m-, o- and p-xylene is 0.33V, 0.75V and 0.93V respectively. In the three isomers, p- and o- xylene are hard to be separated. The possible reason is that the ion mobility coefficient of the two isomers is very closed in high electrical field. The result is very similar with that of in Ref [6]. As the gap distance of drift tube is only 0.2mm, the RF voltage is limited and the compensation voltage is not large. Then the difference between the compensation voltages of xylene isomers is not obvious. But it still proves that the FAIMS system can separate the isomers.

### 3.3 Mass spectra analysis

**Fig 12:**
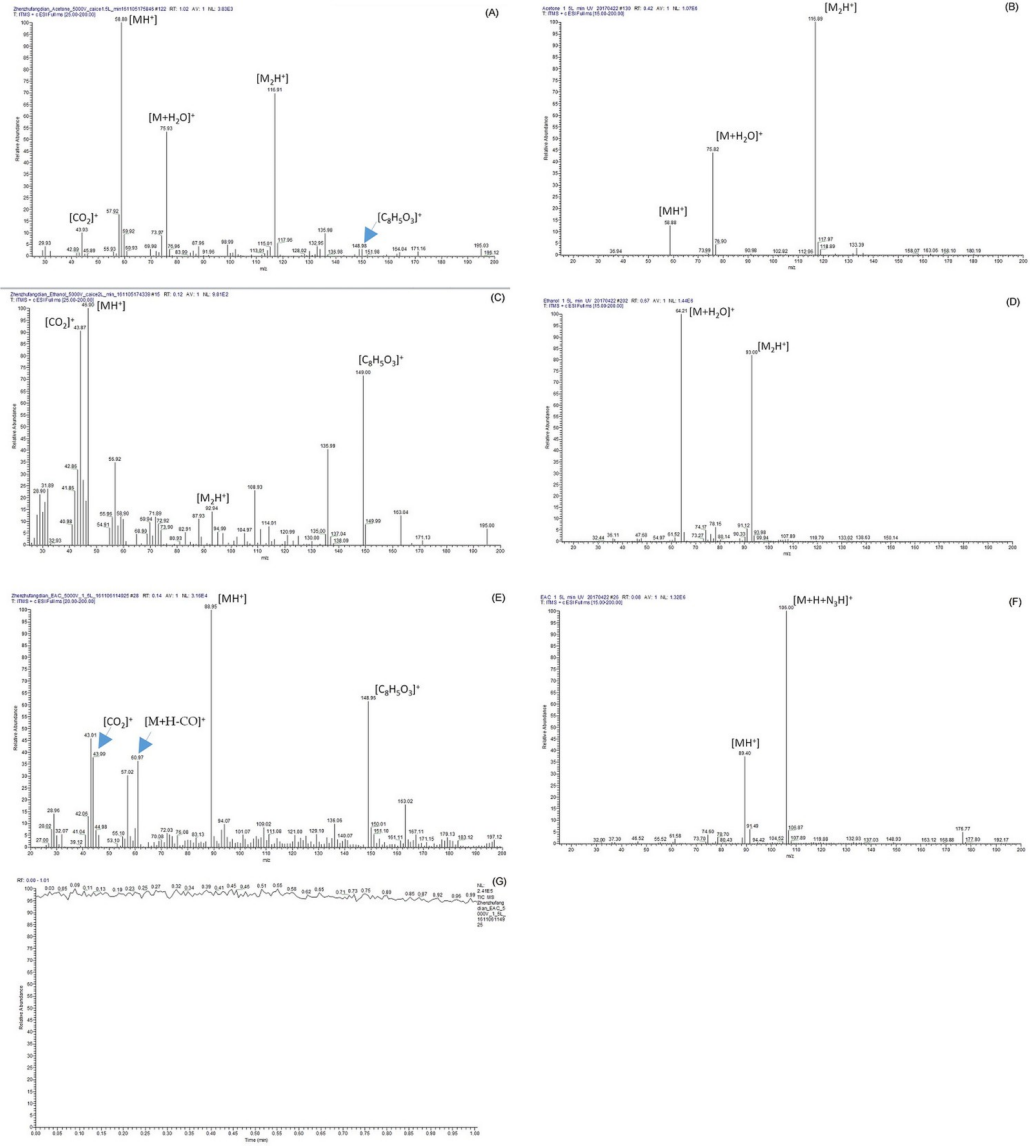
Mass spectra of the needle-to-post and the UV lamp ion sources. (A) Acetone, needle-to-post ion source; (B) Acetone, UV lamp ion source; (C) Ethanol,needle-to-post ion source; (D) Ethanol,UV lamp ion source; (E) Ethyl acetate, needle-to-post ion source; (F) Ethyl acetate, UV lamp ion source; (G) Ethyl acetate, needle-to-post ion source.

Mass spectrometry analysis was performed to understand the types of ions produced by the needle-to-post and UV-lamp ion sources. Mass spectra of the two sources were compared. The samples were the same those used in the FAIMS experiment, the carrier gas flow rate was 1.5 L/min, and the discharge voltage of the needle-to-post ion source was -5 kV. Mass spectra of the two sources are shown in Fig 12.

In Fig 12(A), 12(C) and 12(E) are mass spectra from the needle-to-post ion source, while (B), (D) and (F) are spectra from the ultraviolet lamp ion source. In addition, (h) is stability spectra of the same sample for ionization by the needle-to-post.

In Fig 12A and 12B, the main ion species for acetone ionization were the same. They were protonated ions [CH_3_COCH_3_+H]^+^ (m/z = 59), acetone molecules, and polymerization to form dimer ions [CH_3_COCH_3_+ CH_3_COCH_3_+H]^+^ (m/z = 117) and the polymer of acetone with water [CH_3_COCH_3_+H_2_O]^+^ (m/z = 76) [17]. Because the ionization energy of the needle-to-post ion source was much larger, and the discharge between the needle and post was more intense relative to those parameters for the lamp source, it produced considerable ion debris when ionizing ethanol and ethyl acetate. In the spectrum of ionized ethanol, m/z = 44 was [CO_2_]^+^, and m/z = 47 was the protonated monomer ion [C_2_H_5_OH+H]^+^. For the relatively softer ionization by the UV lamp source, the main ions for the ethanol samples were the polymer of ethanol with water [C_2_H_5_OH+H_2_O]^+^ (m/z = 64) and dimers of ethanol molecules and ions [(C_2_H_5_OH)_2_+H]^+^ (m/z = 93). Similarly, in the ethyl acetate spectra, ions produced by the needle-to-post source were the protonated monomer ion [C_4_H_8_O_2_+H]^+^ (m/z = 89), the ions of m/z = 61[CH_3_CO+H_2_O]^+^ and the ions of m/z = 43 [CH_3_CO]^+^. The ion of m/z = 149[C_8_H_5_O_3_]+ is a background ion produced by phthalate plasticizers. The ions produced by the lamp source were the protonated monomer ion [C_4_H_8_O_2_+H]^+^ (m/z = 89) and the polymer of ethanol with water [C_4_H_8_O_2_+H_2_O]^+^ (m/z = 106).

Fig 12G indicates that the relative abundance stability of the needle-to-post ion source was good, which shows that it can work stably.

The detailed ion information is shown in Table 2.

**Table 2:** Ion species in mass spectra obtained by the needle-to-post electrode.

Gas sample	Molecular weight	Peaks				
		Ionization method	Monomer	Dimer	Cluster	Background ion
acetone	58	The needle	59[MH^+^]*, 44[CO_2_]^+^	117[M_2_H^+^]	76[M+H_2_O]^+^	30,60,74,136,149[C_8_H_5_O_3_]^+^
		The UV lamp	59[MH^+^]	117[M_2_H^+^]*	76[M+H_2_O]^+^	
ethanol	46	The needle	44[CO_2_]^+^ 47[MH^+^]*	93[M_2_H^+^]		29,32,42,43, 74,109,136, 149[C_8_H_5_O_3_]^+^,163,195
		The UV lamp		93[M_2_H^+^]	64[M+H_2_O]^+^ *	
ethyl acetate	88	The needle	44[CO_2_]^+^ 61[M+H-CO]^+^ * 89[MH^+^]			29,43,57,136,149[C_8_H_5_O_3_]^+^,163
		The UV lamp	89[MH^+^]		106[M+H^+^+N_3_H] *	

*The highest peak.

## 4. Discussion

### 4.1 Comparison of FAIMS spectra

In the mass spectra of the needle-to-post and the UV lamp ion sources, the major ions produced from the same sample were similar. Because its ionization energy is larger and the discharge between the needle and post is more intense, there are more ion fragments produced by needle-to-post ion source than by UV lamp ion source.

In the FAIMS detection system, the same compensation voltage corresponds to the same charged ion. The FAIMS experiments were performed with the needle-to-post and UV-lamp ion sources respectively under the same experimental conditions as depicted in Fig 3.

However, because of mounting differences, it is challenging to ensure that the direction and velocity of the carrier gas flow in the drift tube is the same for the two ion sources. Thus, it is difficult to acquire identical FAIMS spectra with the different ion sources.

**Fig 13:**
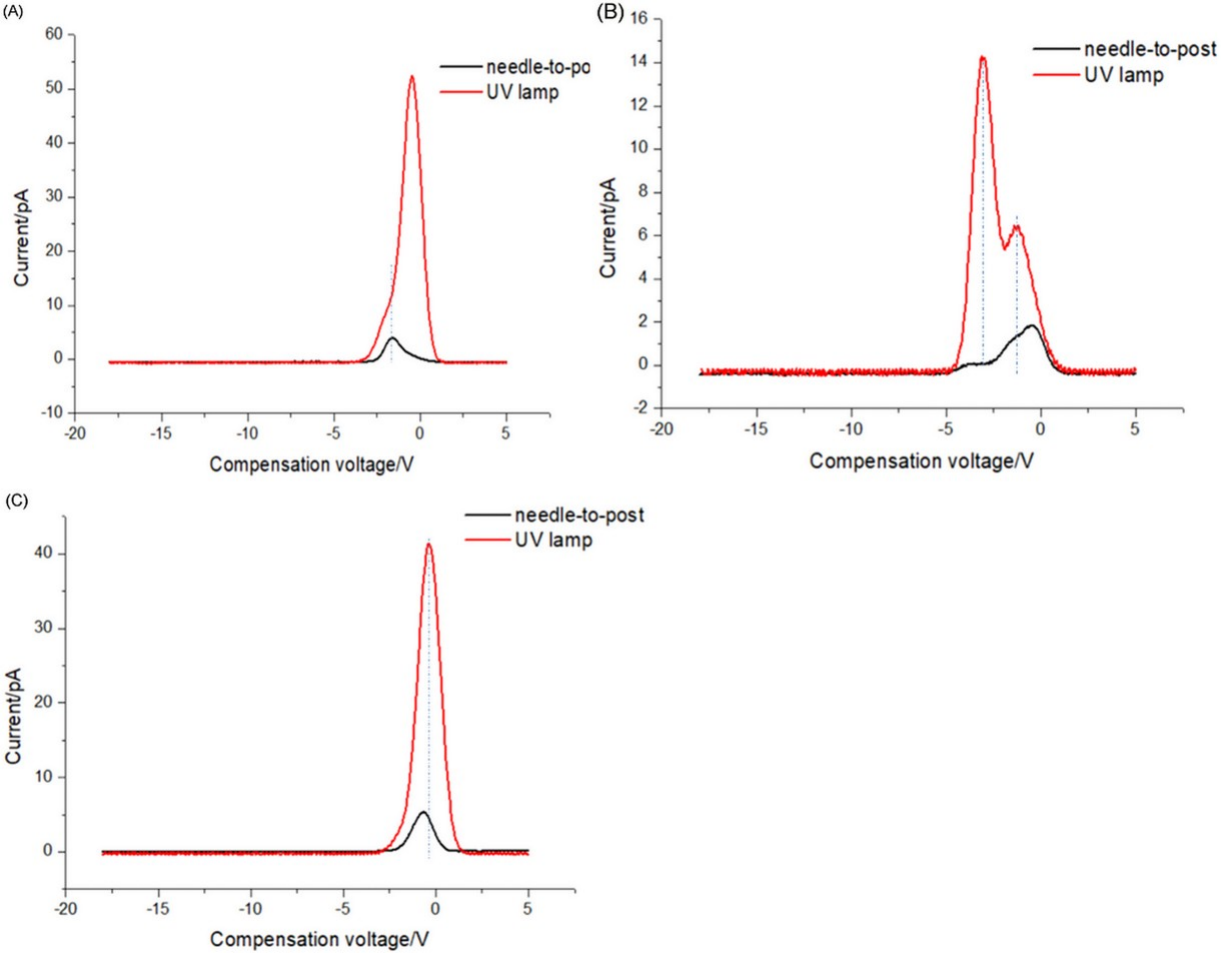
Comparison of FAIMS spectra using the two ion sources. (A) Acetone; (B) Ethanol; (C) Ethyl acetate.

Fig 13 plots FAIMS spectra acquire with the two ion sources, with a 400-V RF voltage and a 1-L/min gas flow. The peak intensity of the needle-to-post ion source was less than that of the lamp because the gas flow hole of UV lamp (∅3mm) was larger than that of the needle-to-post (∅2mm). Thus, more samples were ionized in the lamp source. In addition, there are different ions generated by the two ion sources, which also contributes to the difference in FAIMS spectra. However, almost the same compensation voltage still be acquired with the needle-to-post ion source, even though the peak intensity was less than that of the UV lamp.

### 4.2 The updated FAIMS system

The current FAIMS system is complex and huge, with low integration, poor controllability and low acquisition efficiency. The compensation voltage, the asymmetric waveform generator and controller are separated, which is not convenient for FAIMS experiment.

**Fig 14:**
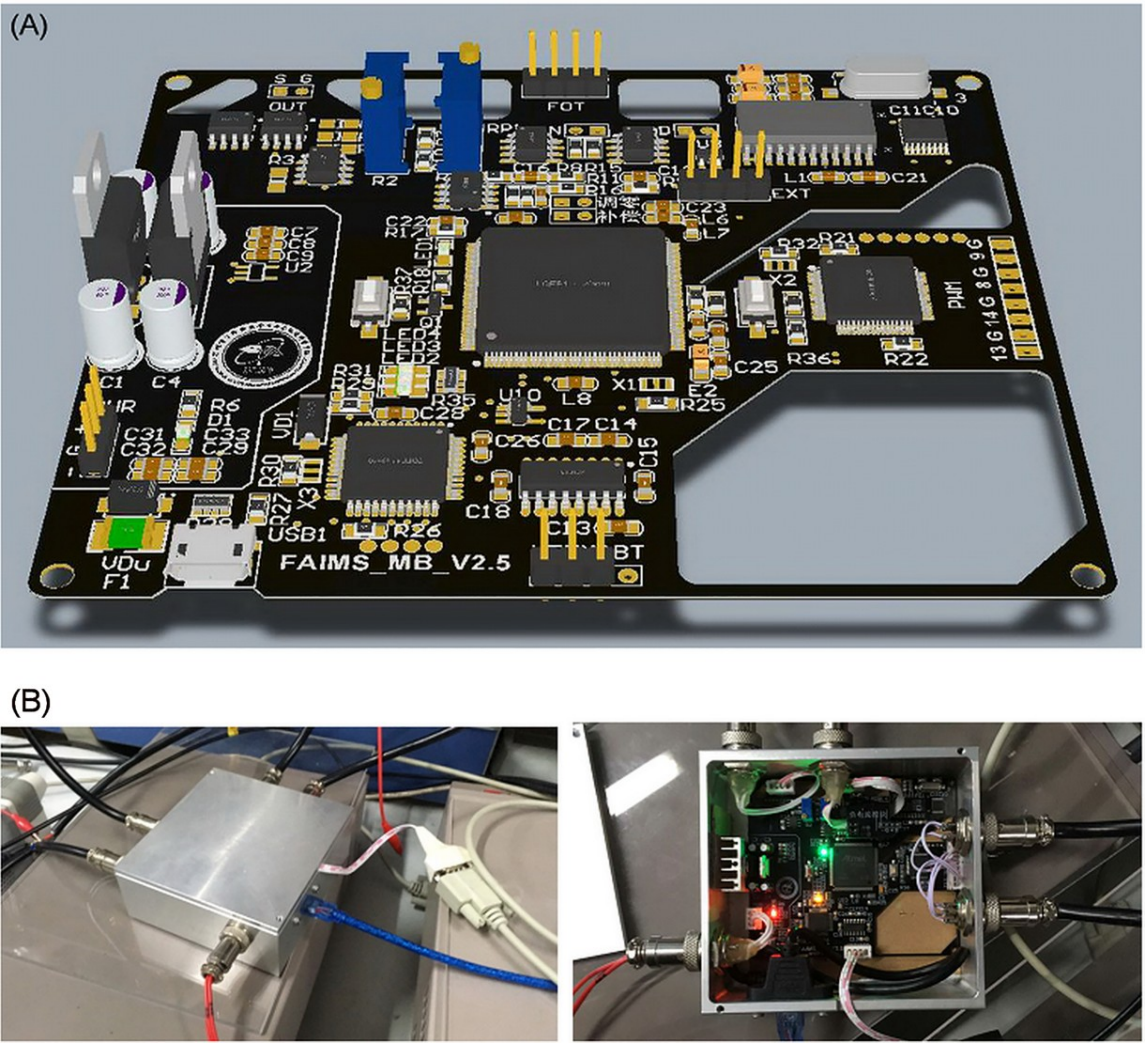
Design of master control board of FAIMS acquisition system. (A) Assembly diagram of the whole machine; (B) 3D rendering diagram of PCB.

In view of the above shortcomings, we intend to design a highly integrated FAIMS master board which integrates the functions of acquisition, control and display, as shown in Fig 14.

The main control consists of the main core, the secondary core, the interface, the acquisition, the feedback control, the isolation and the power supply. The main control board is composed of several embedded chips. It has 24-bit high-precision ADC and 12-bit compensation voltage output. It can also be compatible with multi-front-stage weak current acquisition equipment, including fA current detection chip and Keithley galvanometer. The acquisition process can control the sampling frequency, sampling period and the working range of compensation voltage. At the same time, it can generate two sets of complementary non-phase asymmetric complementary signals to drive the RF power supply. The main control board integrates the function of automatic zero adjustment, range setting and zero baseline adjustment. It also supports multi-platform PC interface, and has Bluetooth wireless transmission function. The size of the main control board is 86×99×32mm^3^. The fully integrated design of the main board reduces the complicated circuit connection.

Also, in the new experimental system, the FAIMS spectra can be draw by QT-based FAIMS gas detection. It realizes the docking between FAIMS system and mobile phone by means of Bluetooth communication in short distance communication. From the user's point of view, based on the purpose of convenience and intuition, combined with the design of FAIMS hardware terminal, the FAIMS spectrum display App is designed and developed in detail. The current and voltage signals of the detected substances can be displayed on the mobile terminal in broken line graphics.

**Fig 15:**
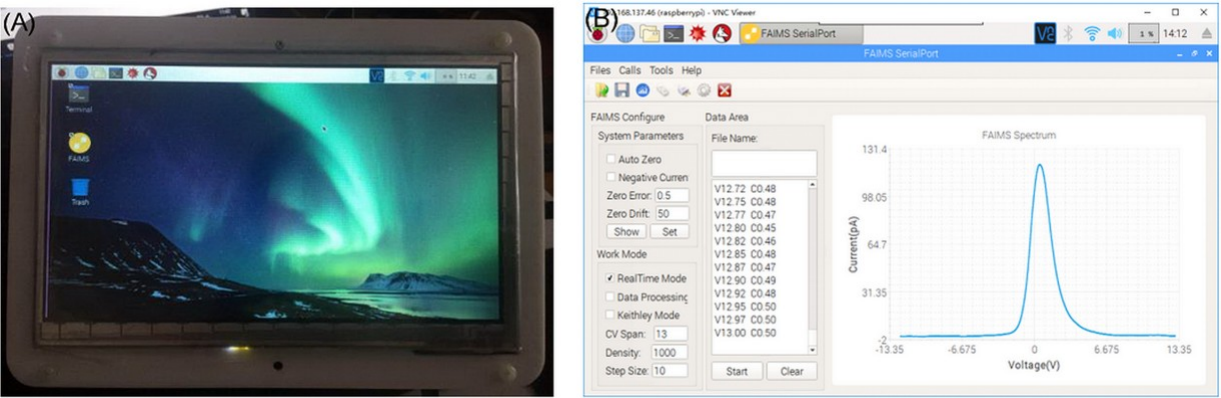
The detection system based on QT. (A) The hardware; (B) The FAIMS spectra.

The QT-based FAIMS gas detection system is a host computer system used in conjunction with the FAIMS lower-level machine main control center. It uses a small power adapter or mobile power supply for power supply, and uses Raspberry Pi 3B embedded open source hardware as a control center (Fig 15).

The mainstream embedded Linux operating system has the characteristics of small size, portable, flexible application and stable operation. This project uses the C++ based QT framework for GUI interface design, which greatly reduces the size and cost of the FAIMS host computer system.

### 4.3 The integrated ion source

**Fig 16:**
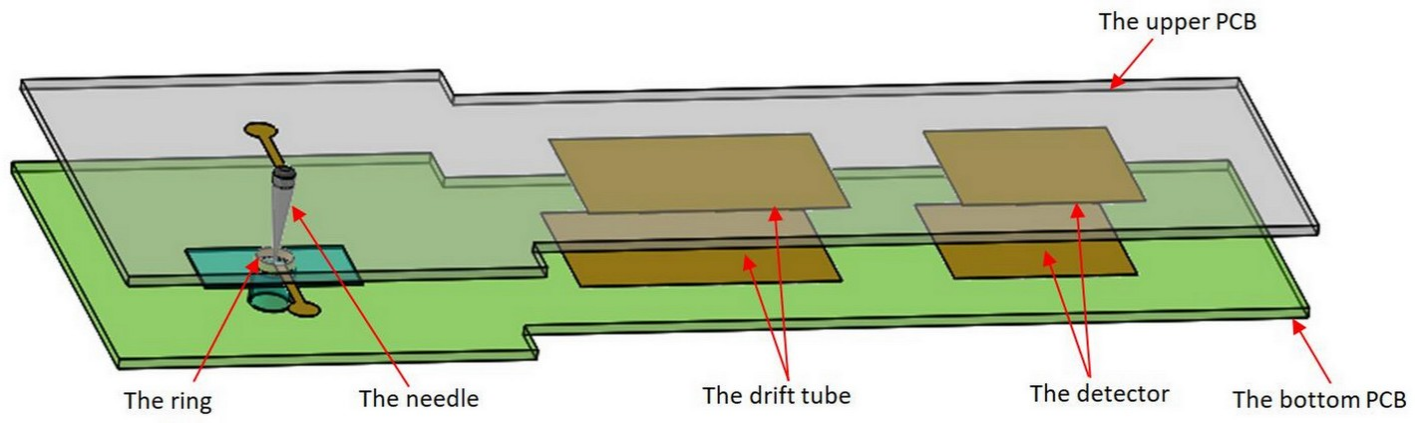
Schematic of integrated FAIMS.

Comparing with the UV lamp ion source, the needle-to-post ion source has many advantages, such as low cost, miniature, simple structure, ease of integration with FAIMS, and so on. On the basis of the needle-to-post, an integrated needle-ring ion source based on PCB technology was also realized recently, as shown in Fig 16.

The needle-ring discharge structure was chosen as the ion source of FAIMS.

**Fig 17:**
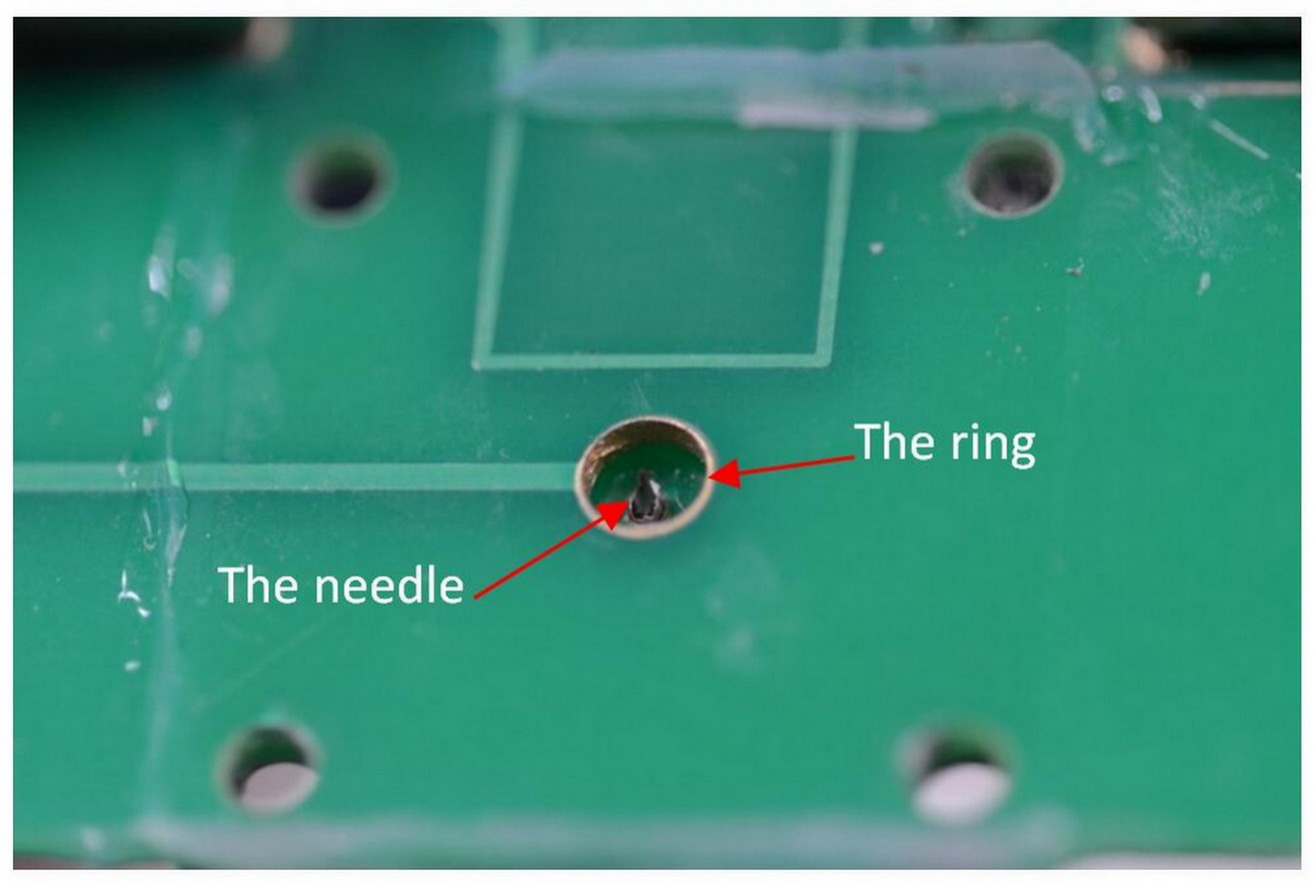
The needle-ring ion source based on PCB.

The photo of the needle-ring ion source is shown in Fig 17.

The needle is welded through a 1mm hole in an upper PCB, and the ring is a copper plating hole with 3mm diameter. Similar with the needle-to-post discharge circuit, the needle is connected with negative voltage and the ring is connected with the ground.

**Fig 18:**
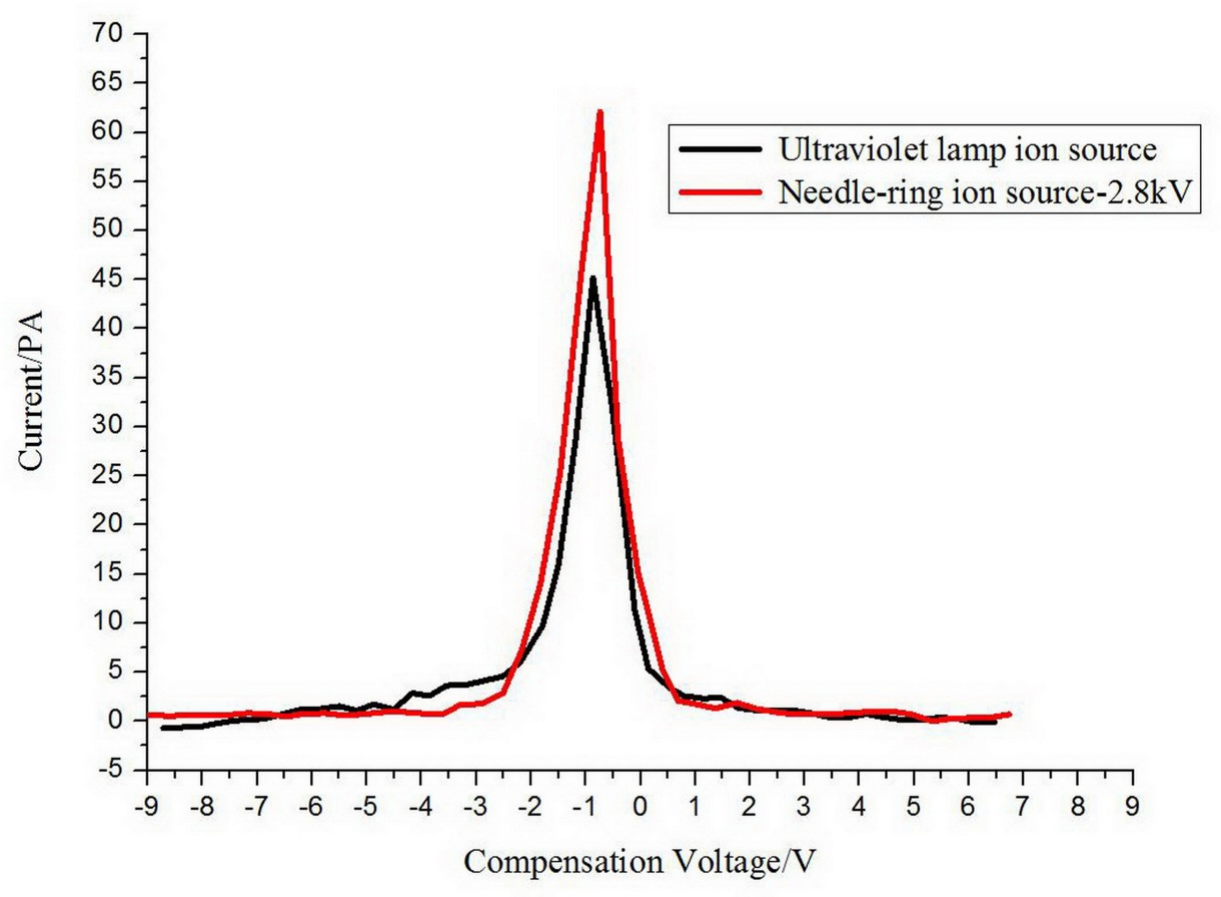
The FAIMS spectra with two ion sources.

The FAIMS spectrum of the acetone is gotten by 300V RF voltage and 1.5L/min gas flow velocity, as shown in Fig 18.

It indicates that the ion intensity is higher for needle-ring ion source than that of UV lamp. It is a promising ion source for the FAIMS and the experimental results in detail will be discussed in the following studies.

### 4.4 Discussion

As an ion source, the designed needle-to-post electrode can ionize the volatile organic compounds and work in tandem with FAIMS. At the same time, it can also produce ionic wind [18–19]. But the ionic wind was not gotten at the exit of FAIMS chip. The possible reason is that the gap distance of the FAIMS is only 0.2mm. Then it needs high pressure for the carrier gas passing through the drift tube. The gas pressure produced by a pair of needle-to-post electrodes is not enough. Then the ionic wind can’t work as the carrier gas. In the future, an array of needle-to-post electrodes are needed, which may replace the sample post. It is the next research subject for the needle-to-post ion source.

## 5. Conclusion

A needle-to-post ion source was successfully used in a FAIMS detection system. Experimental results indicated that the ion source initiates a corona discharge at -4.9 kV, without an external gas supply. With increasing discharge voltage, it enters a glow discharge phase. However, after the carrier gas was introduced, the source directly enters a stable glow discharge phase.

Mass spectrometry experiments comparing the needle-to-post with a UV lamp ion source revealed that the mass spectra were almost the same. The main ion species were protonated monomers and dimers. Because the ionization energy of the post source was larger, there were more ion fragments. In conclusion, the needle-to-post ion source could be used in a FAIMS system instead of a UV lamp ion source.
